# Procalcitonin as a Severity-Based Risk Stratification Marker in Pediatric Infectious Diarrhea: A Prospective Study from the Emergency Department

**DOI:** 10.3390/diagnostics16050662

**Published:** 2026-02-25

**Authors:** Min-Jung Jan, Chun-Yu Chen, Yu-Jun Chang, Wei-Li Liu, Han-Ping Wu

**Affiliations:** 1 Department of Medical Education, Tungs’ Taichung MetroHarbor Hospital, Taichung 435, Taiwan; eric04077@gmail.com; 2 Department of Emergency Medicine, Tungs’ Taichung MetroHarbor Hospital, Taichung 435, Taiwan; yoyo116984@gmail.com; 3 Post-Baccalaureate Medicine, National Chung Hsing University, Taichung 402, Taiwan; 4 Laboratory of Epidemiology and Biostastics, Changhua Christian Hospital, Changhua 500, Taiwan; 83686@cch.org.tw; 5 Department of Pediatrics, Dalin Tzu Chi Hospital, Chiayi County 622, Taiwan; 6 College of Medicine, Chang Gung University, Taoyuan 333, Taiwan; 7 Department of Pediatrics, Chiayi Chang Gung Memorial Hospital, Chiayi County 613, Taiwan

**Keywords:** procalcitonin, pediatric, diarrhea, emergency department

## Abstract

**Objectives**: Procalcitonin (PCT) is used increasingly in emergency settings to guide evaluation of febrile illnesses, but its role in pediatric infectious diarrhea, particularly as a marker of severity, remains unclear. The study objective evaluates whether PCT correlates with clinical severity and outcomes in pediatric infectious diarrhea presenting to the emergency department (ED). **Methods**: This prospective study enrolled 105 children with infectious diarrhea presenting to a tertiary pediatric ED. Serum PCT, C-reactive protein, clinical features, hydration status, treatment decisions (including hospitalization and antibiotic use), and outcomes were analyzed. PCT cutoffs (<0.25, <0.5, and <1.0 ng/mL) were evaluated for their associations with Salmonella infection and severity measures, including dehydration, hospitalization, length of stay, and antibiotic use. **Results**: Thirty-five patients (33.3%) had Salmonella enteritidis. PCT levels did not differ significantly between Salmonella-positive and negative cases (median 0.49 vs. 0.46 ng/mL; *p* = 0.84), and PCT demonstrated poor diagnostic performance (AUC 0.49). In contrast, PCT was strongly associated with markers of severity. Compared with lower PCT levels, children with PCT ≥ 0.25 ng/mL were more frequently hospitalized (92.1% vs. 52.6%; *p* < 0.001) and had longer hospital stays (4.41 vs. 3.00 days; *p* < 0.001). Higher PCT levels were also associated with more dehydration, higher CRP (all *p* < 0.001), and greater antibiotic use (66.7% vs. 23.7%; *p* < 0.001). PCT thresholds of 0.25–0.5 ng/mL consistently identified children at increased risk for admission and higher treatment intensity. **Conclusions**: PCT should not be used as a diagnostic marker for Salmonella enteritidis. Instead, it reflects the host inflammatory response and is strongly associated with clinical severity in children with acute infectious diarrhea evaluated in the ED. The incorporation of PCT thresholds into ED assessment may support early severity-based risk stratification and inform decisions regarding admission and treatment intensity.

## 1. Introduction

Acute infectious diarrhea is one of the most common reasons for pediatric emergency department (ED) visits worldwide. Although most cases are self-limited, a substantial proportion of children develop significant dehydration, systemic inflammation, or complications requiring hospitalization and antibiotic therapy. The early identification of children at higher risk for severe disease remains a major challenge in the ED, where timely triage and appropriate resource utilization are critical.

The clinical assessment of disease severity traditionally relies on symptom duration, hydration status, and overall appearance, yet these features are often nonspecific at initial presentation and may not reliably predict clinical course. Consequently, laboratory biomarkers have been explored as adjunctive tools to support early risk stratification and guide management decisions in the emergency setting. Clinical signs such as poor skin turgor, dry mucous membranes, and prolonged capillary refill are commonly used to assess dehydration; however, these findings have limited sensitivity and inter-observer reliability, particularly in young children, and may not accurately predict subsequent disease severity at initial ED presentation.

Procalcitonin (PCT) is a biomarker of the host systemic inflammatory response and has been widely used in pediatric emergency and critical care settings. Accumulating evidence demonstrates that PCT correlates primarily with disease severity and clinical outcomes rather than with microbiologic or pathogen-specific diagnosis. Consistent findings from pediatric studies in sepsis and lower respiratory tract infections further show that elevated PCT levels are more strongly associated with illness severity, prognosis, and resource utilization than with pathogen identification, supporting its role as a severity-based biomarker applicable beyond sepsis and respiratory infections [1,2,3,4,5].

Adult ED studies of acute infectious diarrhea have similarly shown that PCT has limited diagnostic value for identifying bacterial diarrhea but is associated with disease severity, complications, and adverse outcomes [6,7]. In pediatric populations, prior studies of acute diarrhea have largely focused on etiologic differentiation with inconclusive diagnostic performance [8], whereas ED-based pediatric cohorts, particularly those involving suspected sepsis, have demonstrated associations between elevated PCT levels, hospitalization, and treatment decisions [9]. Collectively, these findings suggest that PCT may reflect disease severity rather than pathogen-specific etiology in pediatric infectious diarrhea.

Despite its widespread clinical use, the role of PCT in pediatric infectious diarrhea remains incompletely defined, particularly in ED-based cohorts. It remains unclear whether PCT can distinguish bacterial pathogens such as *Salmonella enteritidis* from other etiologies and whether its primary clinical value lies in severity assessment rather than diagnosis. Therefore, this prospective study aimed to evaluate the utility of PCT for risk stratification in children presenting to the ED with moderate to severe infectious diarrhea by assessing its diagnostic performance for *Salmonella* infection and its association with clinically relevant severity outcomes, including hospitalization, length of stay, dehydration, inflammatory burden, and antibiotic use.

## 2. Methods

### 2.1. Study Design and Setting

This study was a prospective observational investigation conducted in the pediatric ED of China Medical University Hospital, a tertiary care center in central Taiwan. The study period extended from 1 April 2020 to 31 March 2021. The present analysis represents a prespecified secondary evaluation of a prospectively enrolled cohort that was originally established to examine presentations of children with suspected sepsis and acute infectious diarrhea. For the current investigation, the focus was directed toward the diagnostic performance and severity-assessment utility of serum procalcitonin (PCT) in this population.

### 2.2. Participant Selection

Children were eligible for inclusion if they were between 1 month and 18 years of age and presented with acute diarrheal symptoms characterized by at least three loose or watery stools within a 24 h period. The presence of fever of 38 °C or higher at home or at triage and clinical suspicion of systemic infection or sepsis were required for enrollment. A Vesikari Severity Score of 10 or greater was used to ensure the inclusion of children with at least moderate severity of gastroenteritis. Children were excluded if they had chronic gastrointestinal disease, recent antibiotic exposure within 7 days, a history of international travel within the preceding 14 days, or a final diagnosis inconsistent with infectious enterocolitis, such as urinary tract infection, appendicitis, or intussusception. Patients with mild diarrhea or without fever were not included because this study aimed to evaluate the clinical utility of procalcitonin in children presenting with systemic inflammatory features, where blood testing is clinically justified and early risk stratification is most relevant in the ED. Written informed consent was obtained from the parents or legal guardians of all participants.

### 2.3. Clinical Assessment and Data Collection

Clinical information was collected at the time of ED presentation using a standardized data form. Demographic variables included age and sex. Detailed symptom histories were documented, including the duration of fever, duration and frequency of diarrhea, vomiting, abdominal pain, and the presence of mucus or visible blood in stools. Hydration status was assessed according to World Health Organization clinical criteria, and vital signs were recorded at triage. All data collection was performed by trained research personnel and verified by attending physicians.

### 2.4. Laboratory Evaluation

As part of routine clinical care for suspected infectious enterocolitis or sepsis, all enrolled patients underwent blood and stool testing in the ED. Laboratory assessments included complete blood count with differential, serum PCT, C-reactive protein (CRP), serum electrolytes, and blood glucose. Blood cultures were obtained prior to antibiotic administration when clinically indicated. Stool specimens were cultured for bacterial pathogens, including *Salmonella enteritidis*, *Shigella* spp., *Campylobacter* spp., and diarrheagenic *Escherichia coli*. Salmonella enteritidis was the most prevalent bacterial pathogen identified in this cohort and was the only pathogen with sufficient case numbers to allow meaningful statistical analysis; therefore, pathogen-specific analyses were limited to Salmonella infection. PCT was measured using a standardized chemiluminescent immunoassay platform in the hospital’s central laboratory, with typical result availability within approximately 1 h. PCT levels were measured at emergency department presentation using the Access PCT assay (Beckman Coulter, Inc.; IMMUNOTECH SAS, Marseille, France) on the Access Immunoassay Systems. The assay is a two-step sandwich immunoassay with an analytical range of 0.01–100 ng/mL, automated dilution up to 1000 ng/mL, and a limit of quantitation of ≤0.02 ng/mL. Viral and parasitic pathogens were not routinely tested as part of standard ED evaluation. Clinical management decisions, including fluid resuscitation, antibiotic initiation, and ED observation, were recorded. For admitted patients, the duration of hospitalization was calculated from admission to discharge.

### 2.5. Exposure Definition: Procalcitonin Levels

The principal exposure variable was the serum PCT concentration obtained at ED presentation. PCT was analyzed both as a continuous measurement and as a categorical variable defined by clinically used thresholds of 0.25 ng/mL, 0.5 ng/mL, and 1.0 ng/mL. These cutoffs were selected based on established pediatric infectious disease guidelines and common ED practice for distinguishing low-risk, intermediate-risk, and high-risk bacterial infection [10,11,12].

### 2.6. Outcome Measures

Two major categories of outcomes were evaluated. Salmonella enteritidis positivity was analyzed as a reference bacterial etiology, to examine whether PCT levels differed according to microbiologic findings. Clinical severity outcomes included the need for hospital admission, the total length of hospital stay, the presence of moderate to severe dehydration, the administration and duration of antibiotic therapy, and the inflammatory burden as reflected by CRP levels. Length of stay was analyzed as a continuous variable and additionally evaluated using thresholds of greater than 3 days and at least 5 days to represent clinically meaningful markers of disease severity.

### 2.7. Statistical Analysis

Continuous variables were summarized using medians with interquartile ranges due to non-normal distributions. Group comparisons were conducted using the Mann–Whitney U test when two groups were evaluated, or the Kruskal–Wallis test for comparisons across multiple PCT categories. Categorical variables were compared using the chi-square test or Fisher’s exact test when cell counts were limited. Diagnostic accuracy of PCT for identifying Salmonella infection was assessed by constructing receiver operating characteristic (ROC) curves and calculating the area under the curve (AUC) with corresponding 95% confidence intervals. Given the limited sample size, multivariable modeling for severity outcomes was not performed to avoid model overfitting. Statistical significance was defined as a two-sided *p*-value of less than 0.05. All analyses were conducted using IBM SPSS Statistics version 22.0 (IBM Corp., Armonk, NY, USA).

### 2.8. Ethical Considerations

This study was reviewed and approved by the Institutional Review Board of China Medical University Hospital (No. C1090903010, and date of approval: 26 March 2020). All study procedures adhered to institutional policies and national ethical guidelines for human subjects research. Patient information was de-identified prior to analysis to ensure confidentiality and protection of personal data.

## 3. Results

### 3.1. Baseline Characteristics

A total of 105 children met eligibility criteria and were included in the analysis. Baseline demographic and clinical characteristics did not differ significantly between groups defined by PCT thresholds. As shown in Table 1, children with PCT < 0.25 ng/mL and ≥0.25 ng/mL were similar in age, sex distribution, fever duration, diarrhea duration, stool frequency, and major gastrointestinal symptoms. Hydration status at presentation also did not differ between PCT groups.

### 3.2. PCT and Inflammatory Markers

Across all PCT thresholds, children with elevated PCT had substantially higher CRP levels. In Table 1, the median CRP was significantly higher in the ≥0.25 ng/mL group, and this pattern persisted in Table 2 at the 0.5 ng/mL threshold. This consistency supports a strong association between PCT elevation and systemic inflammatory burden. Stool white blood cell counts exhibited similar trends at lower PCT thresholds, although these associations were less pronounced at higher cutoffs. No significant differences were observed in leukocyte count, neutrophil percentage, hemoglobin, electrolytes, or glucose levels across PCT categories. Outcomes for the PCT ≥ 1.0 ng/mL group were similar to those for the PCT ≥ 0.5 ng/mL group (with comparable hospitalization rates, lengths of stay, and antibiotic use); therefore, a separate table for the ≥1.0 ng/mL threshold was not included to avoid redundancy.

### 3.3. PCT and Hospitalization

**Figure 1:**
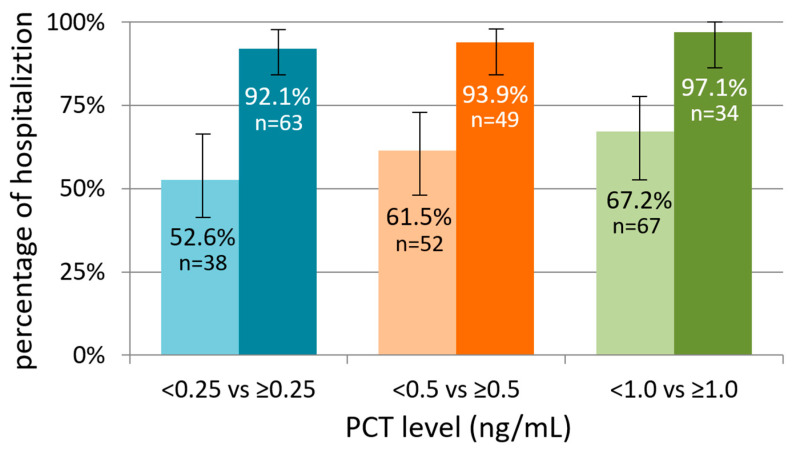
Hospitalization proportion across procalcitonin thresholds. This figure illustrates the percentage of children requiring hospitalization according to three PCT cutoffs (<0.25 vs. ≥0.25 ng/mL, <0.5 vs. ≥0.5 ng/mL, and <1.0 vs. ≥1.0 ng/mL). Error bars represent 95% confidence intervals, and sample sizes for each group are shown above the bars. Across all thresholds, hospitalization frequency increased in higher PCT groups, demonstrating a strong and consistent association between elevated PCT and the need for inpatient care. PCT, procalcitonin.

**Figure 2:**
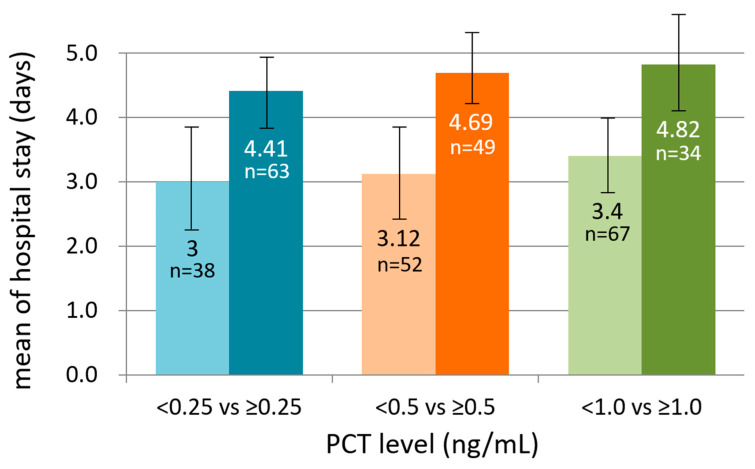
Mean hospital stay across procalcitonin cutoff groups. This figure shows the mean duration of hospitalization (days) for children stratified by PCT thresholds (<0.25 vs. ≥0.25 ng/mL, <0.5 vs. ≥0.5 ng/mL, and <1.0 vs. ≥1.0 ng/mL). Error bars represent 95% confidence intervals, and sample sizes for each group are shown above the bars. A progressive increase in hospital length of stay is observed with rising PCT levels. PCT, procalcitonin.

Elevated PCT was strongly correlated with the need for hospitalization. As shown in Table 1, children with PCT ≥ 0.25 ng/mL were admitted significantly more often than those with lower PCT levels. Table 2 confirms that this association persists with the 0.5 ng/mL threshold. Furthermore, length of hospital stay increased progressively across rising PCT categories. Children with higher PCT levels had longer median hospital stays, and categorical analyses demonstrated that prolonged hospitalization (>3 days or ≥5 days) was more common among children with elevated PCT. These findings indicate that even modest PCT elevation upon ED presentation reflects clinically important severity. As illustrated in Figure 1, hospitalization proportion increased across higher PCT cutoff groups, demonstrating a near stepwise relationship between rising PCT levels and admission frequency. Figure 2 depicts the progressive increase in mean hospital stay across PCT categories, confirming the linear relationship between PCT elevation and prolonged inpatient care.

### 3.4. PCT and Antibiotic Use

Children with higher PCT levels were more frequently treated with antibiotics. Table 1 shows that antibiotic administration was significantly more common in the ≥0.25 ng/mL group, and Table 2 reveals a similar pattern at the 0.5 ng/mL cutoff. Among children receiving antibiotics, those with higher PCT concentrations also required longer courses of therapy. This association aligns with the observed links between PCT, CRP, and hospitalization, further supporting PCT as a marker of clinical severity rather than simple etiologic identification.

### 3.5. PCT and Salmonella Enteritidis

Stool culture identified Salmonella enteritidis in 35 of the 105 children (33%). As shown in Table 3, children in the Salmonella group were significantly younger than those in the non-Salmonella group (mean age: 2.40 vs. 4.43 years, *p* = 0.004), had a shorter duration of fever prior to ED visit (1.86 vs. 2.56 days, *p* = 0.008), and higher platelet counts (305 vs. 266 × 10^3^/µL, *p* = 0.023). There were no significant differences in CRP or PCT levels between the groups (*p* = 0.453 and *p* = 0.842, respectively). PCT and CRP levels did not differ between Salmonella-positive and Salmonella-negative cases, indicating that PCT and CRP do not discriminate microbiologic etiology. As shown in Table 4, the area under the ROC curve (AUC) for detecting Salmonella was 0.545 for CRP and 0.488 for PCT. In predicting hospitalization, the AUCs were similarly low at 0.517 for CRP and 0.476 for PCT. Subgroup analyses based on antibiotic use did not improve discrimination. None of these AUCs reached statistical significance (all *p* > 0.4). Table 5 presents the results of logistic regression analysis for predicting Salmonella enteritidis infection. In the univariate model, younger age, shorter fever duration, higher platelet count, and the presence of gross blood or mucus in stool were significantly associated with Salmonella. In the multivariable model, age ≤ 1 year (adjusted OR 14.6, 95% CI 1.38–154.8, *p* = 0.026), age 2–6 years (adjusted OR 9.48, 95% CI 1.03–87.2, *p* = 0.047), and shorter fever duration (adjusted OR 0.55, 95% CI 0.34–0.89, *p* = 0.014) remained significant predictors. CRP also showed a marginal association (adjusted OR 1.12, 95% CI 1.01–1.24, *p* = 0.040), while PCT was not statistically significant. The presence of gross blood in stool was borderline significant (adjusted OR 2.40, 95% CI 0.89–6.45, *p* = 0.083).

## 4. Discussion

In this prospective ED-based cohort of children with moderate to severe infectious diarrhea, PCT demonstrated a consistent association with disease severity but failed to provide diagnostic discrimination for Salmonella enteritidis infection. These findings clarify the clinical role of PCT in pediatric infectious diarrhea and support its use as a severity-based risk stratification biomarker rather than a pathogen-specific diagnostic test. From an ED perspective, these findings indicate that PCT is better interpreted as an early severity-based risk stratification marker rather than a diagnostic tool for pathogen identification, providing objective support for identifying children at higher risk of requiring intensive management before definitive microbiologic results are available.

Our results are concordant with adult emergency department studies showing that early PCT measurement has limited diagnostic value for identifying bacterial diarrhea but is strongly associated with disease severity, blood stream infection, length of stay, and mortality [6,7]. Similarly, a prior pediatric study in children younger than 5 years demonstrated significantly higher PCT levels in bacterial diarrhea compared with viral diarrhea, but with substantial overlap between bacterial and extraintestinal infections, suggesting that PCT reflects a systemic inflammatory burden rather than pathogen-specific etiology [8]. This observation aligns with our findings that PCT did not reliably distinguish Salmonella infection but was associated with clinical severity and outcomes. The lack of diagnostic discrimination for Salmonella enteritidis was a key finding of this study. Median PCT concentrations were comparable between culture-positive and culture-negative patients, and receiver operating characteristic analysis confirmed diagnostic performance no better than chance. These results reinforce prior evidence that PCT should not be used to infer the etiologic cause of infectious diarrhea and cannot replace stool-based microbiologic testing when bacterial infection is suspected. Instead, PCT appears to capture the host inflammatory response rather than pathogen-specific characteristics.

In contrast, PCT showed strong and clinically meaningful associations with severity-related outcomes. Elevated PCT levels were associated with higher rates of hospitalization, longer length of stay, and increased antibiotic use. These relationships persisted across multiple commonly used thresholds, indicating that even modest elevations in PCT may reflect increased systemic inflammatory burden. This pattern is consistent with pediatric sepsis literature, in which elevated PCT levels and impaired PCT clearance have been associated with illness severity, organ dysfunction, and increased resource utilization rather than pathogen identification alone [1,2,3]. Comparable findings have also been reported in pediatric sepsis and lower respiratory tract infections, where PCT correlates more closely with disease severity and prognosis than with microbiologic diagnosis [4,5]. Although the absolute difference in hospital length of stay between lower and higher PCT groups was approximately 1.4 days, this difference may be clinically meaningful from both patient-care and health-system perspectives. Even a modest reduction in length of stay can translate into improved bed availability, reduced hospitalization-related costs, and decreased caregiver burden, particularly in high-volume pediatric EDs. From an ED triage standpoint, early identification of children at risk for prolonged hospitalization may facilitate more appropriate admission planning and resource allocation.

The observed association between PCT and antibiotic use in this study likely reflects clinician response to perceived disease severity rather than diagnostic accuracy. This underscores the importance of interpreting elevated PCT levels within a severity-based framework. Reliance on PCT as a diagnostic marker for bacterial etiology may lead to inappropriate clinical decisions, whereas its use as an adjunctive severity marker may better support early risk assessment and management planning in the emergency department.

While both PCT and CRP are established markers of systemic inflammation, our findings highlight a distinct clinical profile for PCT in pediatric infectious diarrhea. Although CRP was also significantly elevated in children with higher PCT levels (Table 1 and Table 2), PCT demonstrated stronger and more consistent associations with clinically actionable outcomes, namely hospitalization and antibiotic initiation. This suggests that PCT may more closely reflect the dynamic, host-specific inflammatory burden that prompts clinicians to escalate care, whereas CRP, though correlated, might represent a broader, limited discriminatory ability for key management outcomes, including hospitalization and antibiotic initiation, particularly at the time of ED presentation. In our cohort, PCT thresholds (e.g., ≥0.25–0.5 ng/mL) provided a clearer stratification of patients requiring inpatient management and systemic therapy, underscoring its potential utility as a rapid decision-support tool in the resource-limited ED environment. Importantly, our results indicate that relatively modest elevations in PCT, particularly within the range of 0.25 to 0.5 ng/mL, were consistently associated with higher hospitalization rates and longer hospital stays. These thresholds are commonly used in pediatric emergency practice to differentiate low- and intermediate-risk bacterial infection and may therefore be clinically meaningful for early triage decisions. Rather than serving as a binary diagnostic cutoff, PCT levels within this range may help clinicians identify children who warrant closer monitoring or inpatient management. These findings support the use of PCT as a complementary severity-oriented biomarker, whereas CRP may remain more appropriate for longitudinal inflammatory monitoring rather than immediate triage decisions in pediatric infectious diarrhea. Future comparative studies directly evaluating the incremental value of PCT over CRP in predictive models for admission and antibiotic stewardship would be valuable.

Our findings also extend and refine prior ED-based pediatric research conducted using the same patient source population. A recent prospective study from pediatric emergency departments in Taiwan demonstrated that elevated serum PCT and CRP levels were independently associated with hospitalization and antibiotic use among children with acute infectious diarrhea and suspected sepsis, with Salmonella enteritidis identified as the predominant bacterial pathogen [9]. While that study focused primarily on describing clinical presentations and management decisions in a high-risk cohort defined by suspected sepsis and elevated Vesikari scores, the present analysis specifically evaluated the diagnostic and severity-stratification performance of PCT. By incorporating multiple PCT thresholds, examining associations with hospitalization, length of stay, and antibiotic use, and performing receiver operating characteristic analyses, our study demonstrates that PCT has poor diagnostic performance for Salmonella enteritidis but meaningful value as a marker of disease severity. These additional analyses clarify and extend prior findings by distinguishing severity assessment from etiologic diagnosis.

Accordingly, severity-oriented biomarkers may be particularly valuable in pediatric emergency care. Stool white blood cell counts and culture results were included to explore pathophysiologic associations and outcome correlations, rather than as real-time decision-making tools in the ED, where such results are typically unavailable at initial presentation.

Taken together, our findings support a conceptual shift in the clinical use of PCT in pediatric infectious diarrhea. Rather than serving as a diagnostic marker to differentiate bacterial from viral etiologies, PCT appears to function more effectively as a risk stratification tool reflecting systemic inflammatory burden. This perspective reconciles the inconsistent diagnostic performance reported in prior pediatric diarrhea studies with the more robust prognostic associations observed in severe infections.

Given the strong association between initial PCT levels and hospital course, future research should investigate the prognostic and therapeutic implications of serial PCT measurements in hospitalized children. Dynamic PCT monitoring, tracking changes over 24 to 48 h, might serve as a valuable tool for assessing treatment response, guiding antibiotic duration, and identifying early clinical deterioration or improvement. For instance, a declining PCT trajectory might support earlier de-escalation of antibiotics or discharge planning, whereas a persistent or rising level could signal ongoing inflammation or complications, warranting continued intensive management. Prospective studies evaluating PCT-guided protocols in pediatric infectious diarrhea, similar to those established in sepsis and pneumonia, are needed to determine whether such an approach can improve clinical outcomes, reduce unnecessary antibiotic exposure, and optimize hospital resource utilization.

## 5. Limitations

This study has several limitations that should be considered when interpreting the findings. First, it was conducted at a single tertiary medical center, which may limit generalizability to other EDs with different patient populations or practice patterns. Second, although the cohort was prospectively enrolled, the sample size limited extensive multivariable adjustment, and no formal sample size calculation was performed. Potential seasonal variation cannot be fully excluded, although enrollment was conducted over a full calendar year. Third, because the study population was limited to febrile children with moderate to severe diarrhea, the findings may not be generalizable to children with mild or afebrile diarrheal illness who are commonly managed in outpatient settings. Fourth, PCT was measured at the time of ED presentation, and variation in symptom duration may have influenced biomarker levels; serial measurements were not available to assess dynamic changes. Fifth, stool culture was used as the reference standard for Salmonella enteritidis infection, but its limited sensitivity may have resulted in misclassification of bacterial etiology. Finally, residual confounding related to baseline clinical severity and clinician decision-making cannot be fully excluded. Despite these limitations, the findings provide clinically relevant evidence supporting the role of PCT as a severity marker rather than a diagnostic tool in pediatric infectious diarrhea in the emergency setting.

## 6. Conclusions

In summary, this study demonstrates that PCT should not be used to diagnose Salmonella enteritidis but may serve as an adjunctive biomarker for early severity-based risk stratification in pediatric infectious diarrhea. Future studies should evaluate whether PCT-guided management strategies can improve clinical outcomes, optimize resource utilization, and support antibiotic stewardship in pediatric emergency care.

## Figures and Tables

**Table 1:** Baseline clinical and laboratory characteristics of children with acute infectious diarrhea stratified by procalcitonin < 0.25 ng/mL and ≥0.25 ng/mL.

	PCT								
	<0.25				≥0.25				
	N	Mean	SD	Median	N	Mean	SD	Median	*p*-Value
Age	38	3.58	3.55	2.00	63	3.84	3.53	2.00	0.304
Fever days before PER	38	2.11	1.31	2.00	63	2.46	1.40	2.00	0.137
Maximum Body temperature	38	39.28	0.77	39.40	63	39.45	0.67	39.40	0.350
Diarrhea duration (day)	38	2.08	1.50	1.50	63	2.37	1.45	2.00	0.181
Diarrhea (times/day)	38	6.29	2.66	6.00	63	7.11	3.52	6.00	0.310
WBC	37	9.42	3.76	9.00	63	10.04	4.96	8.60	0.943
Segment (%)	37	58.41	18.26	59.90	63	60.47	16.75	63.00	0.558
Lymphocyte (%)	37	29.70	16.18	28.80	63	26.21	13.05	24.20	0.278
Hemoglobin	37	12.20	1.04	12.40	63	12.13	1.00	12.20	0.858
Platelet	37	302.00	105.11	267.00	63	264.24	67.63	269.00	0.177
CRP	38	2.42	2.02	1.83	63	8.06	5.82	6.32	<0.001
PCT	38	0.15	0.07	0.17	63	4.59	9.57	1.12	<0.001
Na	38	136.26	2.01	136.00	63	135.22	2.96	136.00	0.109
K	38	4.14	0.51	4.10	63	4.16	0.50	4.20	0.702
Glucose	37	97.65	11.85	98.00	63	101.75	21.06	99.00	0.340
Lactate	29	14.48	8.51	12.80	32	12.85	4.87	11.55	0.660
Stool routine (WBC)	31	7.45	24.81	0.00	58	13.59	28.61	0.00	0.040
Duration of antibiotic therapy	9	4.22	1.99	4.00	42	4.64	2.10	4.00	0.469
Fever subsided after admission (days)	20	2.65	2.08	2.00	58	2.41	1.64	2.00	0.910
Observation stay (hour)	18	5.00	3.09	3.50	5	6.00	1.41	5.00	0.226
Hospital stay (day)	38	3.00	2.60	3.00	63	4.41	2.16	4.00	<0.001

CRP, C-reactive protein; PCT, procalcitonin; PER, pediatric emergency room; WBC, white blood count.

**Table 2:** Comparison of clinical and laboratory characteristics according to procalcitonin < 0.5 ng/mL and ≥0.5 ng/mL.

	PCT								
	<0.5				≥0.5				
	N	Mean	SD	Median	N	Mean	SD	Median	*p*-Value
Age	52	3.33	3.15	2.00	49	4.18	3.87	3.00	0.131
Fever days before PER	52	2.21	1.45	2.00	49	2.45	1.29	2.00	0.163
Maximum Body temperature	52	39.36	0.78	39.40	49	39.42	0.63	39.30	0.837
Diarrhea duration (day)	52	2.13	1.58	1.00	49	2.39	1.34	2.00	0.102
Diarrhea (times/day)	52	6.75	3.58	6.00	49	6.86	2.85	6.00	0.498
WBC	51	9.37	3.72	9.00	49	10.26	5.27	8.60	0.801
Segment (%)	51	58.55	16.62	59.90	49	60.91	17.99	63.00	0.504
Lymphocyte (%)	51	29.24	14.59	27.30	49	25.69	13.93	24.10	0.195
Hemoglobin	51	12.14	1.15	12.40	49	12.17	0.86	12.20	0.959
Platelet	51	294.59	95.75	269.00	49	261.16	68.95	257.00	0.135
CRP	52	2.64	1.97	2.40	49	9.44	5.85	7.85	<0.001
PCT	52	0.21	0.13	0.21	49	5.79	10.57	1.45	<0.001
Na	52	136.12	1.93	136.00	49	135.08	3.24	136.00	0.175
K	52	4.19	0.48	4.10	49	4.12	0.52	4.20	0.600
Glucose	51	96.82	14.76	98.00	49	103.78	20.84	100.00	0.161
Lactate	38	14.13	7.70	12.55	23	12.78	5.17	11.20	0.542
Stool routine (WBC)	44	8.11	25.30	0.00	45	14.71	29.16	1.00	0.033
Duration of antibiotic therapy	15	4.00	1.65	4.00	36	4.81	2.20	4.00	0.226
Fever subsided after admission (days)	32	2.50	2.00	2.00	46	2.46	1.59	2.00	0.500
Observation stay (hour)	20	5.15	3.00	4.50	3	5.67	1.15	5.00	0.487
Hospital stay (day)	52	3.12	2.42	3.00	49	4.69	2.16	4.00	<0.001

**Table 3:** Comparison of clinical features and biomarkers between Salmonella-positive and Salmonella-negative enteritidis.

	Salmonella Enteritidis								
	(−)				(+)				
All	N	Mean	SD	Median	N	Mean	SD	Median	*p*-Value
Age	70	4.43	3.98	3.00	35	2.40	1.74	2.00	0.004
Fever days before PER	70	2.56	1.43	2.00	35	1.86	1.06	2.00	0.008
Maximum Body temperature	70	39.45	0.70	39.40	35	39.21	0.68	39.00	0.122
Diarrhea duration (day)	70	2.41	1.56	2.00	35	2.06	1.33	2.00	0.277
Diarrhea (times/day)	70	6.66	2.94	6.00	35	7.43	3.84	6.00	0.438
WBC	69	9.66	4.95	8.40	34	9.89	3.56	9.10	0.251
Segment (%)	69	59.79	18.42	61.70	34	57.97	15.54	58.10	0.715
Lymphocyte (%)	69	27.76	15.47	25.40	34	27.85	12.47	24.95	0.913
Hemoglobin	69	12.17	1.07	12.30	34	12.25	0.94	12.30	0.966
Platelet	69	266.30	81.40	259.00	34	305.21	84.77	293.50	0.023
CRP	70	5.67	5.40	3.64	35	5.94	5.64	4.41	0.453
PCT	68	2.40	6.16	0.46	33	3.99	10.53	0.49	0.842
Na	70	135.89	2.53	136.00	35	135.20	2.91	135.00	0.197
K	70	4.08	0.52	4.05	34	4.30	0.42	4.25	0.053
Glucose	69	101.20	19.66	98.00	34	98.00	14.40	99.00	0.902
Lactate	43	13.10	7.31	11.20	21	14.32	5.34	13.20	0.155
Stool routine (WBC)	57	12.11	28.98	0.00	35	9.69	23.60	0.00	0.339
Duration of antibiotic therapy	37	4.32	2.07	4.00	15	5.07	2.02	5.00	0.134
Fever subsided after admission (days)	52	2.21	1.47	2.00	28	2.86	2.14	2.00	0.337
Observation stay (hour)	18	5.39	3.01	5.00	7	4.57	1.90	5.00	0.689
Hospital stay (day)	70	3.50	2.11	4.00	35	4.46	2.84	4.00	0.128

CRP, C-reactive protein; PCT, procalcitonin; PER, pediatric emergency room; WBC, white blood count.

**Table 4:** Diagnostic performance of C-reactive protein and procalcitonin for Salmonella enteritidis and for hospitalization among children with acute infectious diarrhea.

		Overall						Hospitalization (+)					
Tests	Group	Area	SE	95% CI			*p*-Value	Area	SE	95% CI			*p*-Value
CRP	All	0.545	0.056	0.436	-	0.655	0.453	0.517	0.065	0.390	-	0.643	0.805
	Antibiotic (−)	0.656	0.074	0.510	-	0.802	0.059	0.644	0.103	0.442	-	0.847	0.188
	Antibiotic (+)	0.508	0.083	0.345	-	0.671	0.928	0.511	0.083	0.348	-	0.674	0.901
PCT	All	0.488	0.062	0.367	-	0.609	0.842	0.476	0.071	0.338	-	0.614	0.729
	Antibiotic (−)	0.568	0.084	0.402	-	0.733	0.424	0.572	0.111	0.354	-	0.789	0.519
	Antibiotic (+)	0.521	0.101	0.323	-	0.720	0.816	0.522	0.101	0.324	-	0.720	0.812

Area, Area Under the ROC Curve; CRP, C-reactive protein; PCT, procalcitonin; SE, Salmonella enteritidis.

**Table 5:** Univariate and multivariable analyses of factors associated with Salmonella enteritidis in children with acute infectious diarrhea.

			Salmonella (+)		Univariate Analysis					Multiple Analysis (Adjusted)				
		Total	N	%	Odds Ratio	95% CI			*p*-Value	Odds Ratio	95% CI			*p*-Value
Age	≤1	21	11	52.4	16.500	1.832	-	148.606	0.012	14.629	1.383	-	154.773	0.026
	2–6	68	23	33.8	7.667	0.952	-	61.715	0.056	9.478	1.030	-	87.187	0.047
	≥7	16	1	6.3	1.000					1.000				
Fever days before PER	Median (IQR)	2 (1–3)	2 (1–2)		0.617	0.417	-	0.913	0.016	0.545	0.335	-	0.887	0.014
Platelet	Median (IQR)	269 (228–330)	294 (244–345)		1.006	1.000	-	1.011	0.033					
CRP	Median (IQR)	3.82 (1.84–7.64)	4.41 (2.53–7.25)		1.009	0.937	-	1.087	0.805	1.117	1.005	-	1.241	0.040
PCT	Median (IQR)	0.49 (0.20–1.36)	0.49 (0.20–0.99)		1.024	0.974	-	1.078	0.350					
Gross blood in stool	No	63	14	22.2	1.000					1.000				
	Yes	42	21	50.0	3.500	1.499	-	8.170	0.004	2.400	0.893	-	6.448	0.083
Gross mucus in stool	No	45	9	20.0	1.000									
	Yes	60	26	43.3	3.059	1.255	-	7.458	0.014					
Antibiotics	No	53	20	37.7	1.000					1.000				
	Yes	52	15	28.8	0.669	0.295	-	1.515	0.335	0.327	0.105	-	1.018	0.054

## Data Availability

The datasets used and/or analyzed during the current study are available from the corresponding author on reasonable request.

## Abbreviations

AUC	area under the curve
CRP	C-reactive protein
ED	emergency department
OR	odds ratio
PCT	procalcitonin
ROC	receiver operating characteristic
